# Efficacy of Thermal Ablation for Treatment of High-Grade Cervical Dysplasia Among HIV-Positive Women: Preliminary Results From Western Kenya

**DOI:** 10.1200/GO.22.40000

**Published:** 2022-05-05

**Authors:** Chemtai Mungo, Cyrillus Ogollah, Jennifer Ambaka, Jackton Omoto, Craig Cohen

**Affiliations:** ^1^ University of North Carolina Chapel Hill, Chapel Hill, NC; ^2^ Kenya Medical Research Institute (KEMRI)/FACES, Nairobi, Kenya; ^3^ Family AIDS Care and Education Services (FACES) Clinic, Kisumu, Kenya; ^4^ Maseno University School of Medicine, Maseno, Kenya; ^5^ University of California, San Francisco, San Francisco, CA

## Abstract

In 2019, the World Health Organization (WHO) endorsed thermal ablation (TA) for use within “screen-and-treat” cervical cancer prevention programs in low- and middle-income countries (LMICs), including among women living with HIV (WLWH). We evaluated TA efficacy for treatment of biopsy-confirmed cervical intraepithelial neoplasia grades 2 and 3 (CIN2/3) among WLWH in western Kenya (ClinicalTrials.gov NCT04191967).

**METHODS:**

Between August 2019 and November 2020, WLWH age 25-65 years underwent high-risk human papillomavirus (hrHPV) self-collection. hrHPV-positive women underwent colposcopy-directed biopsies, and thermal ablation treatment if eligible per WHO guidelines. Women with biopsy-confirmed CIN2/3 had colposcopy-directed biopsies at 12-months to determine treatment efficacy.

**RESULTS:**

Sixty-eight hrHPV-positive WLWH with biopsy-confirmed CIN2/3 at baseline; 14 CIN2, 54 CIN3, underwent thermal ablation. Mean age and parity were 41.2 years and 4, respectively. The mean CD4 count was 473.98 cells/mm3 and 96.9% had HIV viral suppression. Fifty-eight women (83.8%) have been seen for a 12-month follow-up visit, and pathology results are available for 54 (79.4%). Of these, 35 (66.0%) had successful treatment, defined as biopsy-confirmed CIN1 or normal findings 12-months following treatment, while 18 (34.0%) had treatment failure - persistent biopsy-confirmed CIN2/3. Treatment failure was 23.1% 95% CI (13.0 to 45.9) and 37.5%, 95% CI (22.1 to 52.0) among women with CIN2 and CIN3 at baseline, respectively.

**CONCLUSION:**

Hand-held thermal ablation devices are affordable, portable, easy to use, and hence highly scaleable within screen-and-treat programs in LMICs. However, our preliminary results, with rigorous disease status verification at both baseline and follow-up find higher than previously reported treatment failure rates for CIN3 among WLWH, a high-risk population for cervical cancer. If replicated by larger studies, this highlights a potential limitation of the current WHO cervical cancer elimination strategy, calling for better risk stratification in this population, and/or consideration of adjuvant therapy to prevent CIN2/3 recurrence following thermal ablation.

